# Supplementary material for: Contamination of 8.2 ka cold climate records by the Storegga tsunami in the Nordic Seas
Source: Nat Commun. 2024 Apr 4;15:2904. doi: 10.1038/s41467-024-47347-9 (PMC10995125; doi:10.1038/s41467-024-47347-9)
Supplement: Supplementary file 1 — Supplementary Information [file 41467_2024_47347_MOESM1_ESM.pdf]

Supplementary Information for:

Contamination of 8.2 ka cold climate records by the Storegga tsunami in the Nordic Seas.

Stein Bondevik[1], Bjørg Risebrobakken[2], Steven J. Gibbons[3], Tine L. Rasmussen[4] and Finn Løvholt[3]

1 Department of Civil Engineering and Environmental Sciences, Western Norway University of Applied Sciences, P.O. Box 133, N-6851 Sogndal, Norway, stein.bondevik@hvl.no.

2 NORCE Climate & Environment, Bjerknes Centre for Climate Research, Bergen, Norway, bjri@norceresearch.no.

3 Norwegian Geotechnical Institute (NGI), P.O. Box. 3930 Ullevål Stadion, N-0806 Oslo, Norway, steven.gibbons@ngi.no; finn.lovholt@ngi.no.

4 Department of Geosciences, UiT the Arctic University of Norway, Tromsø, Norway, tine.rasmussen@uit.no.

Contents

Supplementary Fig. 1| Simulation of wave height and flow speed near Storegga ... 2
Supplementary Fig. 2| Simulation of maximum surface elevation of the Storegga tsunami ... 3
Supplementary Fig. 3| Simulation of maximum flow velocity of the Storegga tsunami ... 4
Supplementary Table 1| Overview of the sediment cores ... 5
Supplementary Table 2| Critical velocity by Storegga tsunami for erosion at core sites ... 5

Supplementary Methods 1. Age calibration and age–depth models ... 6
1.1 Re-calibration of the age of the Storegga tsunami event ... 6
1.2 Calibration of radiocarbon ages and reservoir age correction ... 6
1.3 Age-depth models for sediment cores using BACON ... 6

Supplementary Methods 2. Age-depth models for sediment cores ... 7
2.1 Age-depth model for sediment core MD95-2011, Vøring plateau ... 7
Supplementary Fig. 4| Age–depth model 1 of core MD95-2011 ... 7
Supplementary Fig. 5| Age–depth model 2 of core MD95-2011 ... 8
2.2 Age model for sediment core LINK 14, east of the Faroe Islands ... 9
Supplementary Table 3| Ages used in the age-depth modelling of core LINK14 ... 9
Supplementary Fig. 6| Age–depth model 1 for core LINK 14 ... 10
Supplementary Fig. 7| Age–depth model 1 for core LINK 14 ... 11
2.3 Age–depth models of sediment core 28-03 in the Norwegian channel ... 12
Supplementary Fig. 8| Age–depth model 1 for core Troll 28-03 ... 12
Supplementary Fig. 9| Age–depth model 2 for core Troll 28-03 ... 13

Supplementary References ... 14

Supplementary Fig. 1| Simulation of wave height and flow speed near Storegga

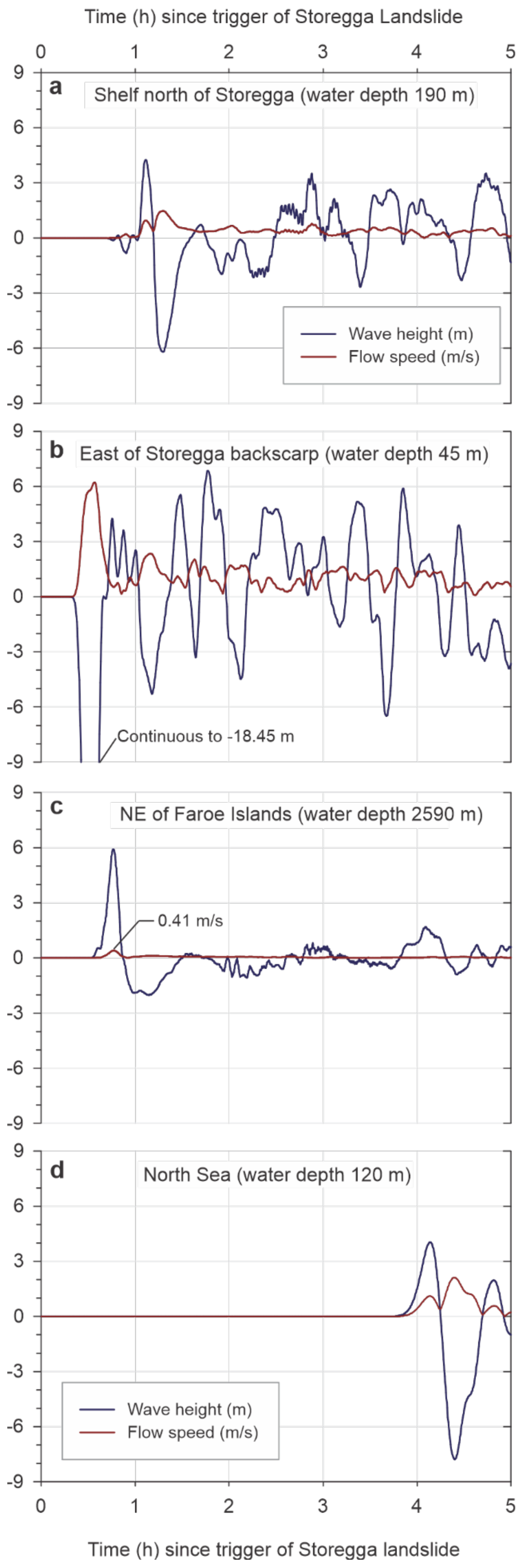

Supplementary Fig. 1| Simulation of wave height and flow speed down to about 1–10 m above the sea floor at locations surrounding Storegga. The y-axis is the same for all curves. See map in Fig. 1 (article) for locations. **a** Norwegian shelf north of Storegga. **b** Between Storegga and Western Norway. **c** Northeast of Faroe Islands. **d** North Sea. Paleo-water depths in brackets.

Supplementary Fig. 2| Simulation of maximum surface elevation of the Storegga tsunami

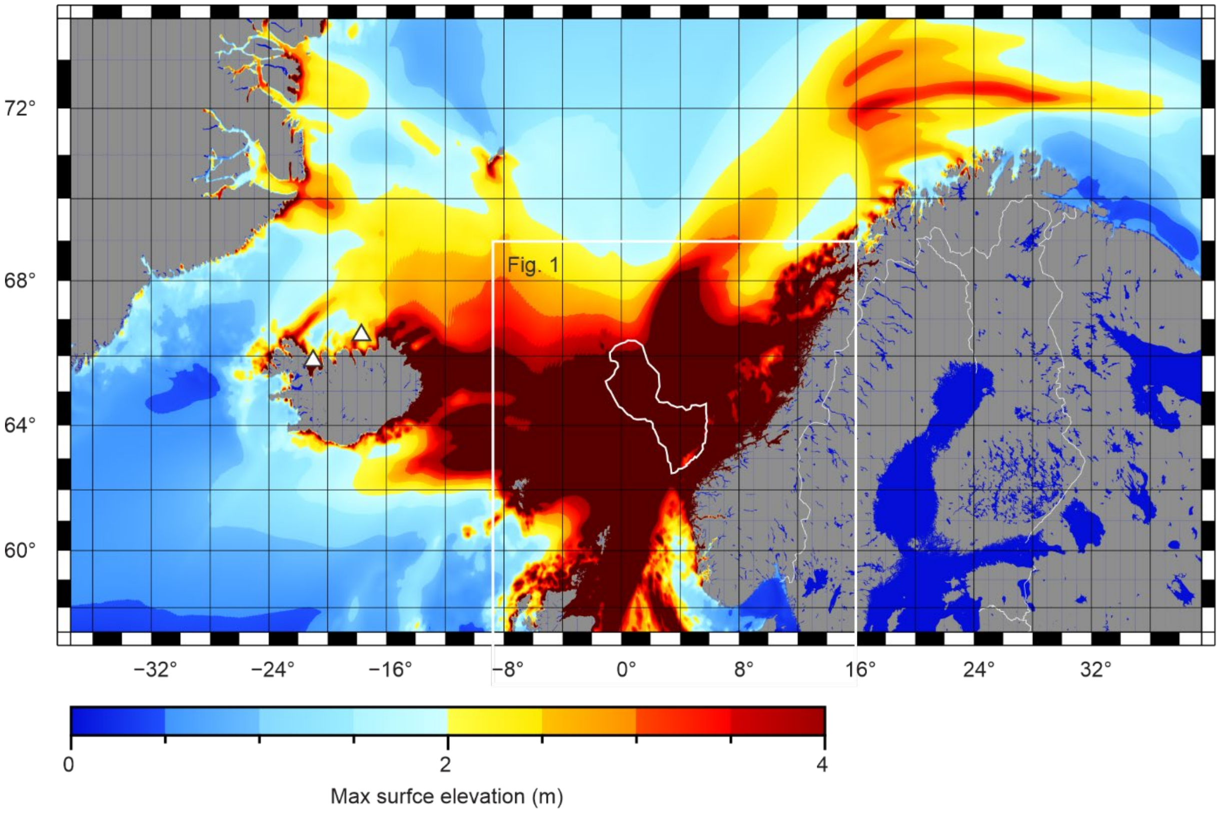

Supplementary Fig. 2| Extended simulation of the Storegga tsunami in the Nordic Seas showing maximum surface elevation (wave amplitude). Pixels in dark red brown have surface elevation > 4 m. White square shows the extent of Figure 1 in article. White outline of the Storegga Slide shows the run out of slide debris. White triangles are locations of sediment cores north of Iceland with possible Storegga tsunami disturbance. In the westernmost core there is evidence of redeposited Neogene coccoliths [1], the easternmost core shows a peak of the epifaunal foraminifera *Cibicides lobatulus* [2], both could possibly be caused by redeposition of sediments eroded by the Storegga tsunami. The simulation was done with the present-day bathymetry.

Supplementary Fig. 3| Simulation of maximum flow velocity of the Storegga tsunami

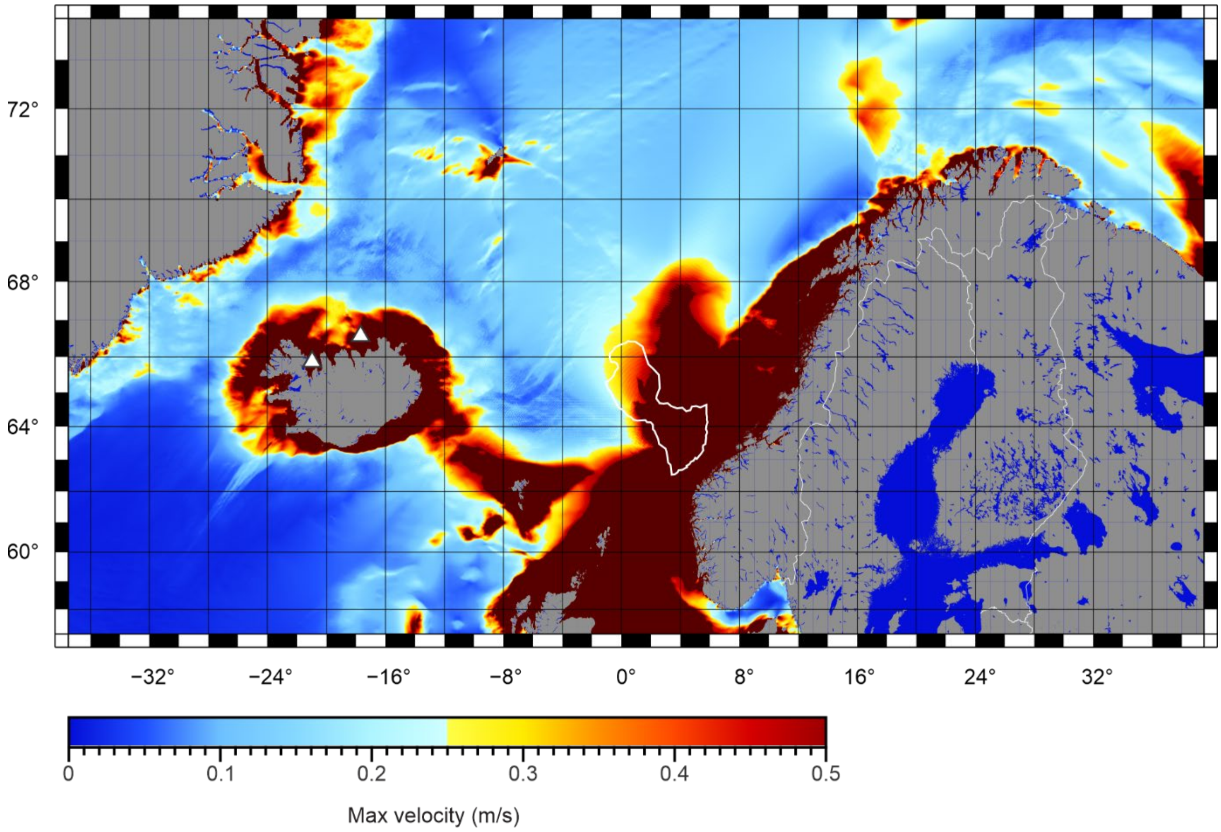

Supplementary Fig. 3. Maximum flow velocity of the Storegga tsunami down to about 1–10 m above the sea floor. Pixels in red brown color have maximum velocity > 0.5 m/s. Sediment cores north of Iceland with possible Storegga tsunami disturbance in white triangles. In the westernmost core there is evidence of redeposited Neogene coccoliths [1], the easternmost core shows a peak of the epifaunal foraminifera *Cibicides lobatulus* [2], both could possibly be caused by redeposition of sediments eroded by the Storegga tsunami. The simulation was done with the present-day bathymetry.

Supplementary Table 1| Overview of the sediment cores

Core	Latitude	Longitude	Water depth	Paleo water depth[§]	Core length	Core-depth of «8.2 ka layer»
MD95-2011[*]	66.969° N	7.639° E	1048 m	965.3 m	745 cm	533–535 cm
28-03[†]	60.867° N	3.733° E	345 m	321.8 m	475 cm	340–345 cm
LINK14[#]	61.717° N	5.823° W	346 m	279 m	565 cm	109–116 cm

[*] Risebrobakken, et al. [3]
[†] Klitgaard-Kristensen, et al. [4]; Klitgaard-Kristensen, et al. [5]
[#] Rasmussen and Thomsen [6]
[§] Depths read out of the grid used in the simulations –accounts for changes in bathymetry since ca. 8150 cal yr BP; Hill, et al. [7]

Supplementary Table 2| Critical velocity by Storegga tsunami for erosion at core sites

Site	Max. uniform velocity in water column	Velocity 1 m above sea floor*	Grain size eroded Sundborg[†]		Miller et al., 1977
			Smallest grain[#]	Largest grain	Largest grain[§]
LINK14	1.77 m/s	1.24 m/s	0.01 mm	5 mm	5.6 mm
28-03	0.68 m/s	0.54 m/s	0.06 mm	0.9 mm	0.6 mm
MD95-2011	0.38 m/s	0.36 m/s	–	0.2 mm	0.15 mm

[*] Estimated, velocity based on simulations by Williams and Fuhrman [8] of boundary layer.
[†] Grain size limits read off the Sundborg diagram [9] for flows one meter above sea bed.
[#] Consolidated sediments (silt and clay).
[§] Grain size calculated from equations in Miller, et al. [10] for flows one meter above sea bed.

Supplementary Methods 1. Age calibration and age–depth models

1.1 Re-calibration of the age of the Storegga tsunami event

We have tested the original calibration of the radiocarbon ages of the green moss fragments [11], picked out of the tsunami deposits and believed to have been killed the day the tsunami happened[12], with IntCal20 [13] and the sequence function in OxCal v.4.4 [14]. We found only a minor change compared with the original calibration in Bondevik, et al. [11].

i. The unmodelled date of the mean of the green moss ages of 7300 ±20 ^{14}C years BP is 8030–8180 cal BP (95.4 % level) – the same as in Bondevik, et al. [11].

ii. The modelled date of the mean of the green moss ages of 7300 ±20 ^{14}C years BP with the sequence function, including radiocarbon ages above and below the Storegga tsunami deposits, is **8080–8180 cal BP** (95.4 % level) – this changed by 10 years from 8070–8180 cal yr BP in Bondevik, et al. [11].

iii. The mean of the modelled date of the green moss age is 8139 cal yr BP with 1 σ = 27 yr. This could be written as 8140 ± 55 cal yr BP (2 σ range).

1.2 Calibration of radiocarbon ages and reservoir age correction

We used a ΔR value of −145 ± 35 years and the Marine20 calibration curve [15] to correct the radiocarbon ages to calendar years. The ΔR value for the open Norwegian Sea and North Sea was found from the radiocarbon ages of a group of 19 whales, as recommended by Bondevik and Gulliksen in Mangerud, et al. [16]. This ΔR value of −145 ± 35 years corresponds to 7 ± 11 years relative to the Marine04 calibration curve). We have used http://calib.org/marine/ (last accessed 2023/12/06) to calculate the ΔR value relative to the Marine20 calibration curve.

1.3 Age-depth models for sediment cores using BACON

To produce the age–depth models for the sediment cores we used the program BACON, version 2.3.9.1 [17] installed as a package in the statistical software called R [18]. For each of the cores we performed two models: Model 1 includes all or most of the dates (specified in each case below) with no prior assumptions. In Model 2 we used the options *hiatus.depths=* the lower boundary of the 8.2 ka layer and *slump=* the thickness of the 8.2 ka layer, and discarded dates found within the layer that could be redeposited. We placed the *hiatus* depth right below the layer, allowing for a gap or break in the sedimentation, and the *slump* at the depths of the layer allowing for an instantaneous sedimentation between the given depths. Documentation for the Bacon program is found at (https://chrono.qub.ac.uk/blaauw/manualBacon_2.3.pdf, last accessed 2023/12/06)

Supplementary Methods 2. Age-depth models for sediment cores

2.1 Age-depth model for sediment core MD95-2011, Vøring plateau

We used all the published ages from the core [19] except the redeposited ages from within the 8.2 ka layer, and included our three new ages from just above and just below the 8.2 ka layer (Table 1 in article) – in all 32 ages of planktonic foraminifera. In addition, we also included the Vedde Ash Bed, dated to 12,121 ± 57 ice core years [20]. In the first simulation (Supplementary Fig. 4) we had no prior assumptions (model 1).

Supplementary Fig. 4| Age–depth model 1 of core MD95-2011

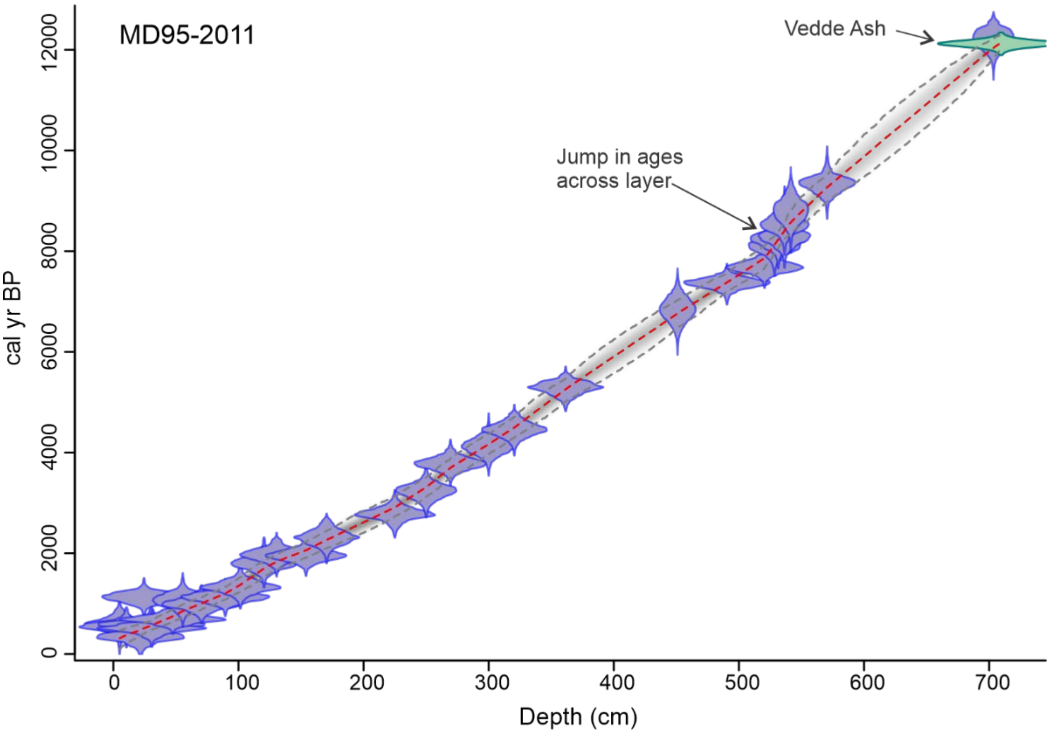

Supplementary Fig. 4| Age–depth model 1 of core MD95-2011. In all 32 radiocarbon ages, as described above have been included. We see a distinct jump in ages across the depth of the 8.2 ka yr sand layer. This could indicate a hiatus at this level.

In model 2 we used the same ages as in model 1, but we assumed that the sediments between 533 cm and 535 cm depth were redeposited and that there were a hiatus at 535 cm. (Commands in R were written as **slump = c(533, 535)** and **hiatus.depth=535**.)

Supplementary Fig. 5| Age–depth model 2 of core MD95-2011

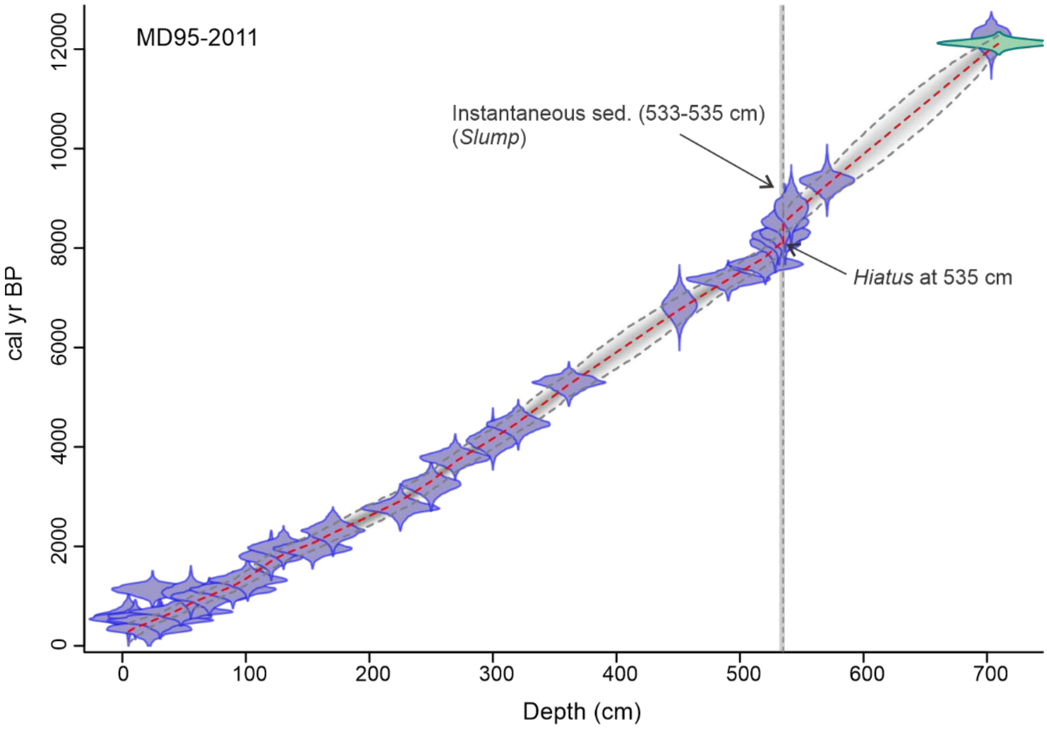

Supplementary Fig. 5| Age–depth model 2 of core MD95-2011. Here we assume instantaneous sedimentation ("*slump*") between 533 and 535 cm and a hiatus at 535 cm. The modelled hiatus has a mean of 400 years and corresponds to about 20 cm of erosion. Sedimentation rates through the core varies between 15 and 21 yr/cm or 0.5–0.7 mm/yr.

We conclude that age model 2 is the better model for the time around the 8.2 ka event and use it – the red stippled curve in Supplementary Fig. 5 – as the age-depth model to plot the different proxies of the core in Fig. 3 of the article. The jump in ages across the sand layer is an additional indication of re-sedimentation and erosion at the core site at this level.

According to age model 2 the upper boundary of the sand layer at 533 cm is 7831–8382 (95.4 % level) with mean of 8097 years. The hiatus has a mean of 395 years (257–531 years) – this corresponds to about 20 cm of erosion beneath the sand layer.

2.2 Age model for sediment core LINK 14, east of the Faroe Islands

According to Rasmussen and Thomsen [6], a sand layer is present between 114 and 116 cm depth – inferred to be deposited by the Storegga tsunami. We used the ages as published [6] and added the three new dates of foraminifera, at depths of 108, 114 and 117 cm (Supplementary Table 3).

Supplementary Table 3| Ages used in the age-depth modelling of core LINK14

**Lab.no**	**Depth (cm)**	**Species**	**^{14}C age BP**	**Cal yr BP (2 σ)**
AAR-6841	10	*Bivalve*	525 ± 40	
AAR-8143	35	*H. balthica*	3885 ± 150	
AAR-8144	75	*C. laevigata*	6295 ± 65	
UB-18949	108	*C. laevigiata*	7856 ± 42	
UB-18950	114	*C. laevigiata*	8140 ± 36	
UB-18951	117	*C. laevigiata*	8354 ± 37	
AAR-8145	140	*C. laevigata*	8640 ± 90	
Saksunarvatn Ash	185			10,297 ± 45*
AAR-6842	195	*Bivalve*	9700 ± 80	

The AAR-lab no. were published in Rasmussen and Thomsen [6]. The UB-lab no. dated at the ^{14}Chrono Centre at the Queen's University Belfast.
* GRIP age of the Saksunarvatn Ash [20,21].

Model 1 includes all the dates in Supplementary Table 3 and has no prior assumptions. We notice a slight «bump» - upward convex age-depth curve across the depth of the sand layer in question that could indicate re-sedimentation and older ages (Supplementary Fig. 6). The age of the upper boundary of the sand layer at 114 cm depth is in this model 8566 (8397–8749) cal yr BP, about 400 years older than the age of the Storegga green moss.

Supplementary Fig. 6| Age–depth model 1 for core LINK 14

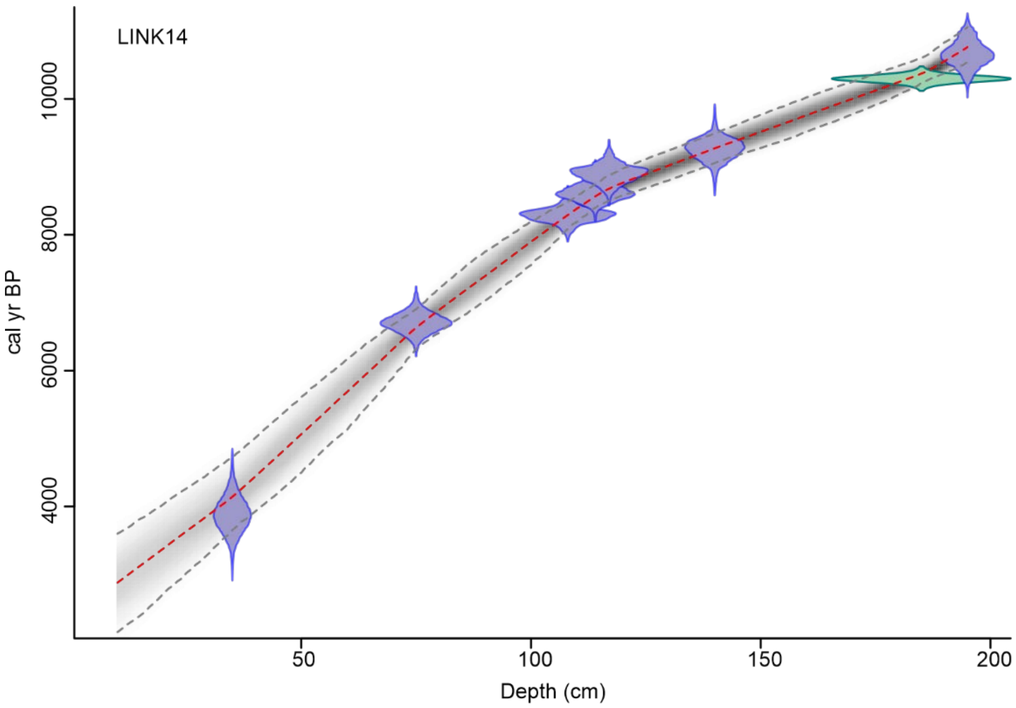

Supplementary Fig. 6| Age–depth model 1 for core LINK14 east of the Faroe Islands. There is a «bump» in the age–depth curve across the sand layer. In green color is the GRIP age of the Saksunarvatn Ash Bed (Supplementary Table 3).

Model 2 excludes the date within the sand layer, at 114 cm, and includes a hiatus at 116 cm depth and instantaneous sedimentation between 109 and 116 cm (*slump*). According to the grain size diagram of the sediment core, Figure 7 in Rasmussen and Thomsen [6], the sediments are fining upwards in terms of a decreasing percentage of sand from 114 upwards to 109 cm. We thus place the upper boundary of the layer at 109 cm.

We used the following code for this modelling: > Bacon(«LINK14», 2, hiatus.depths=116, slump=c(109,116))

The mean of the upper boundary of the layer is in this simulation 8224 cal yr BP, about 100 years older than Storegga, but on a 2-σ-range the age (7929–8465 cal yr BP) falls well within the time frame of the Storegga tsunami.

Supplementary Fig. 7| Age–depth model 1 for core LINK 14

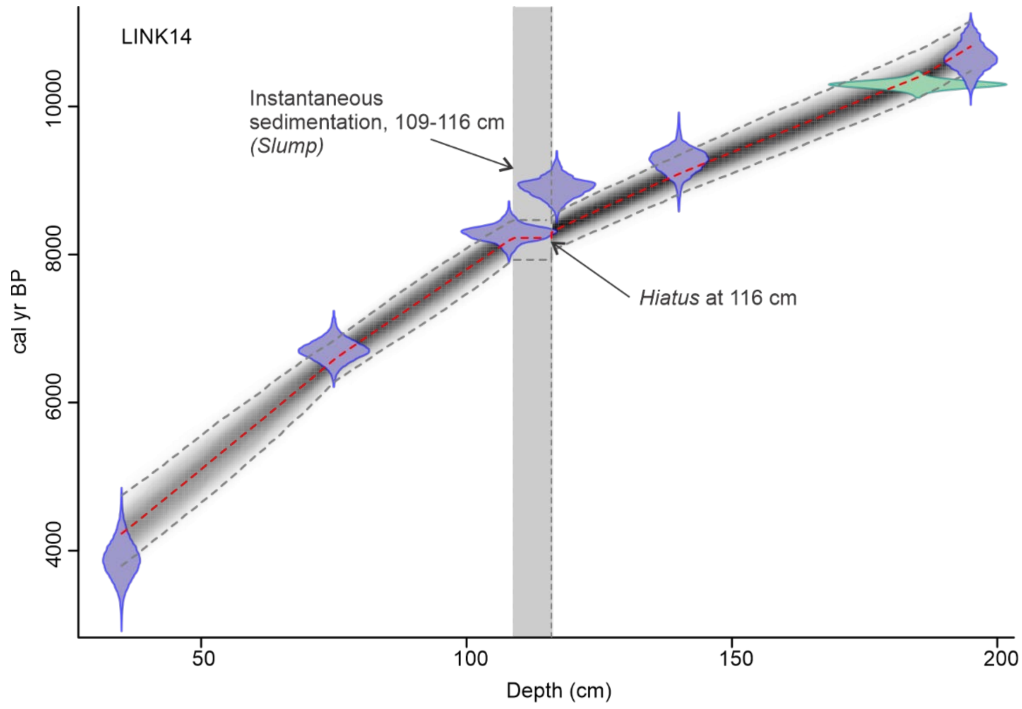

Supplementary Fig. 7| Age-depth model 2 of core LINK14. Here we assume a hiatus at 116 cm and instantaneous sedimentation (*slump*) between 109 and 116 cm. The modelled hiatus is 68 years, corresponding to about 2 cm. Age of the upper boundary of the layer at 109 cm is 8224 (7929–8465) cal yr BP.

2.3 Age–depth models of sediment core 28-03 in the Norwegian channel

We used the nine radiocarbon ages as published in Table 2 in Klitgaard-Kristensen, et al. [4] for core 28-03. The core is 475 cm long, covers the last 10,000 years and the 8.2 ka layer is located between 341 and 344 cm depth. The layer consists of finer grained material than above it and below it in the core.

Supplementary Fig. 8| Age–depth model 1 for core Troll 28-03

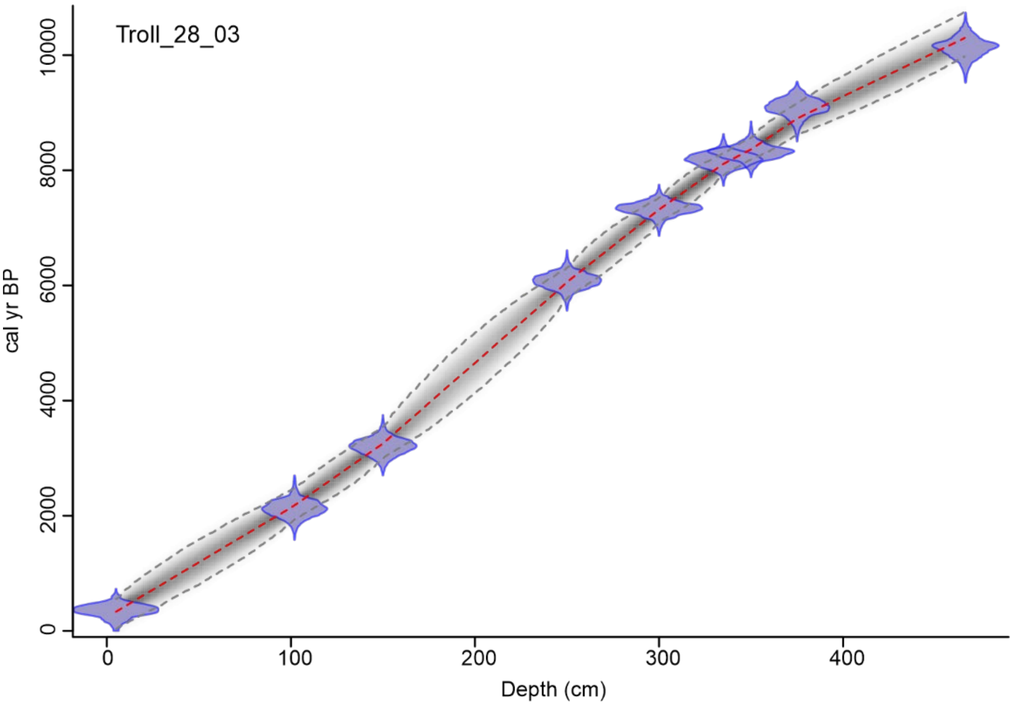

Supplementary Fig. 8| Age–depth model 1 for core Troll 28-03. Model 1 has no prior assumptions. The modelled age of the upper boundary of the layer is 8214 (8029–8413) cal yr BP.

Model 2 includes a hiatus at the lower boundary of the layer at 344 cm and instantaneous sedimentation (slump) between 341–344 cm (Supplementary Fig. 9). The modelled hiatus is 100 years, corresponding to 5 cm of erosion. The age of the upper boundary of the layer at 341 cm is 8188 (7934–8411) cal yr BP.

Supplementary Fig. 9| Age–depth model 2 for core Troll 28-03

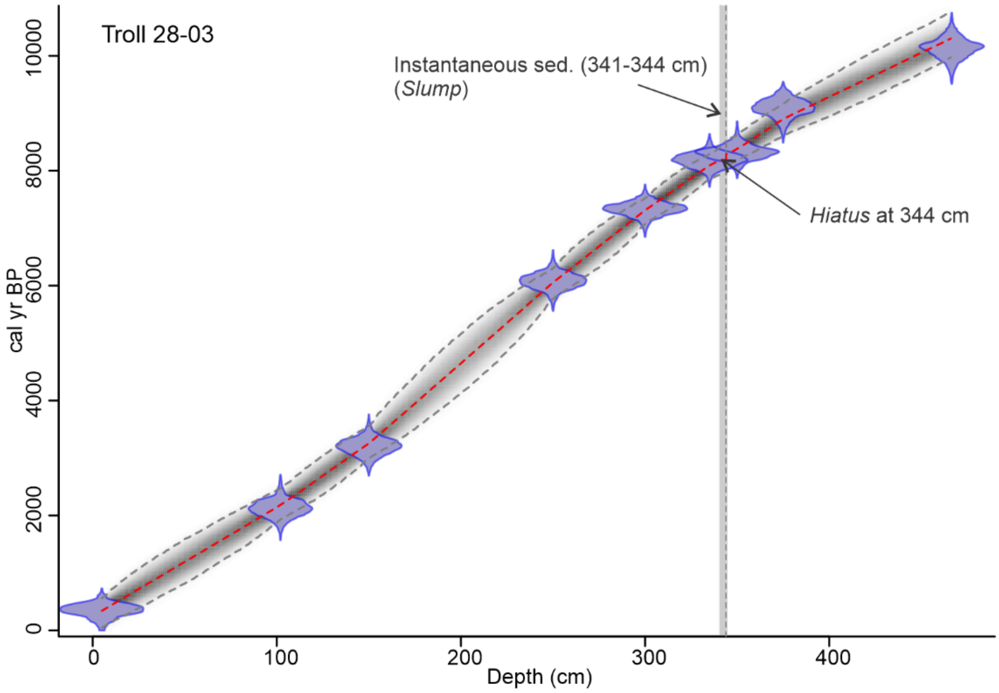

Supplementary Fig. 9| Age–depth model 2 for core Troll 28-03. Here we assumed a hiatus at 344 cm and instantaneous sedimentation (*slump*) between 341 and 344 cm. The modelled hiatus is 100 years corresponding to about 5 cm of erosion. Sedimentation rater varies from 0.4–0.7 mm/yr. Mean of the upper boundary of the layer is 8188 cal yr BP.

The two different age models for core 28-03 are not very different and we cannot say whether model 1 or model 2 is the best model. According to age model 2 there could be a short hiatus below the layer.

Supplementary References

1 Andrews, J. T. & Giraudeau, J. Multi-proxy records showing significant Holocene environmental variability: the inner N. Iceland shelf (Húnaflói). *Quaternary Science Reviews* **22**, 175-193 (2003).

2 Knudsen, K. L., Søndergaard, M. K. B., Eiríksson, J. & Jiang, H. Holocene thermal maximum off North Iceland: Evidence from benthic and planktonic foraminifera in the 8600–5200 cal year BP time slice. *Marine Micropaleontology* **67**, 120-142 (2008).

3 Risebrobakken, B., Jansen, E., Andersson, C., Mjelde, E. & Hevrøy, K. A high-resolution study of Holocene paleoclimatic and paleoceanographic changes in the Nordic Seas. *Paleoceanography* **18** (2003).

4 Klitgaard-Kristensen, D., Sejrup, H. P. & Haflidason, H. The Last 18 kyr Fluctuations in Norwegian Sea Surface Conditions and Implications for the Magnitude of Climatic Change: Evidence from the North Sea. *Paleoceanography* **16** (2001).

5 Klitgaard-Kristensen, D., Sejrup, H. P., Haflidason, H., Johnsen, S. & Spurk, M. A regional 8200 cal. yr BP cooling event in northwest Europe, induced by final stages of the Laurentide ice-sheet deglaciation? *Journal of Quaternary Science* **13**, 165-169 (1998).

6 Rasmussen, T. L. & Thomsen, E. Holocene temperature and salinity variability of the Atlantic Water inflow to the Nordic Seas. *The Holocene* (2010).

7 Hill, J., Collins, G. S., Avdis, A., Kramer, S. C. & Piggott, M. D. How does multiscale modelling and inclusion of realistic palaeobathymetry affect numerical simulation of the Storegga Slide tsunami? *Ocean Modelling* **83**, 11-25 (2014).

8 Williams, I. A. & Fuhrman, D. R. Numerical simulation of tsunami-scale wave boundary layers. *Coastal Engineering* **110**, 17-31 (2016).

9 Sundborg, Å. The River Klarälven a Study of Fluvial Processes. *Geografiska Annaler* **38**, 125-316 (1956).

10 Miller, M. C., McCave, I. N. & Komar, P. D. Threshold of sediment motion under unidirectional currents. *Sedimentology* **24**, 507-527 (1977).

11 Bondevik, S., Stormo, S. K. & Skjerdal, G. Green mosses date the Storegga tsunami to the chilliest decades of the 8.2 ka cold event. *Quaternary Science Reviews* **45**, 1-6 (2012).

12 Rydgren, K. & Bondevik, S. Moss growth patterns and timing of human exposure to a Mesolithic tsunami in the North Atlantic. *Geology* **43**, 111-114 (2015).

13 Reimer, P. J. *et al.* The IntCal20 Northern Hemisphere Radiocarbon Age Calibration Curve (0–55 cal kBP). *Radiocarbon* **62**, 725-757 (2020).

14 Bronk Ramsey, C. Bayesian Analysis of Radiocarbon Dates. *Radiocarbon* **51**, 337-360 (2009).

15 Heaton, T. J. *et al.* Marine20—The Marine Radiocarbon Age Calibration Curve (0–55,000 cal BP). *Radiocarbon* **62**, 779-820 (2020).

16 Mangerud, J., Bondevik, S., Gulliksen, S., Karin Hufthammer, A. & Høisæter, T. Marine 14C reservoir ages for 19th century whales and molluscs from the North Atlantic. *Quaternary Science Reviews* **25**, 3228-3245 (2006).

17 Blaauw, M. & Christen, J. A. Flexible paleoclimate age-depth models using an autoregressive gamma process. *Bayesian Analysis* **6**, 457-474, 418 (2011).

18 R: A language and environment for statistical computing. R Foundation for Statistical Computing (Vienna, Austria 2022).

19 Eldevik, T. *et al.* A brief history of climate – the northern seas from the Last Glacial Maximum to global warming. *Quaternary Science Reviews* **106**, 225-246 (2014).

20 Rasmussen, S. O. *et al.* A new Greenland ice core chronology for the last glacial termination. *Journal of Geophysical Research: Atmospheres* **111** (2006).

21 Lohne, Ø. S., Mangerud, J. & Birks, H. H. Precise 14C ages of the Vedde and Saksunarvatn ashes and the Younger Dryas boundaries from western Norway and their comparison with the Greenland Ice Core (GICC05) chronology. *Journal of Quaternary Science* **28**, 490-500 (2013).
